# Effectiveness, safety and health-related quality of life of multiple sclerosis patients treated with fingolimod: results from a 12-month, real-world, observational PERFORMS study in the Middle East

**DOI:** 10.1186/s12883-017-0913-3

**Published:** 2017-08-07

**Authors:** Anat Achiron, Hany Aref, Jihad Inshasi, Mohamad Harb, Raed Alroughani, Mahendra Bijarnia, Kathryn Cooke, Ozgur Yuksel

**Affiliations:** 1 Multiple Sclerosis Center, Sheba Medical Center, 52621 Tel-Hashomer, Israel; 2 Ain Shams University, Cairo, Egypt; 3 Rashid Hospital and Dubai Medical College, Dubai, UAE; 4 Department in Monla Hospital, Tripoli, Lebanon; 5 Division of Neurology, Department of Medicine, Amiri Hospital, Sharq, Kuwait; 6 Novartis Healthcare Pvt. Ltd., Hyderabad, India; 7 Novartis Pharma AG, Basel, Switzerland

**Keywords:** Real-world, Observational, Fingolimod, Other DMTs, Health-related quality of life, Effectiveness, Safety, PERFORMS

## Abstract

**Background:**

Evidence on the use of fingolimod in real-world clinical practice and data on patient-reported health-related quality of life (HRQoL) in countries such as the Middle East are sparse. The Prospective Evaluation of Treatment with Fingolimod for Multiple Sclerosis (PERFORMS) study assessed HRQoL and effectiveness and safety of fingolimod in patients with relapsing-remitting multiples sclerosis (RRMS), primarily in Middle Eastern countries.

**Methods:**

This 12-month, observational, multicentre, prospective, real-world study was conducted in patients with RRMS who initiated fingolimod or another approved disease-modifying treatment (DMT) within 4 weeks before study entry. Patients were enrolled in a 2:1 ratio to obtain more data in fingolimod and parallel in other DMTs cohort by physicians during routine medical care. Key study outcomes included HRQoL assessed using MS International QoL (MusiQoL), MS relapses and disability. Safety was assessed throughout the study period. Due to the observational nature of the study, no neuroimaging assessments were mandated and central reading was not performed.

**Results:**

Of 249 enrolled patients, 247 were included in the analysis (fingolimod cohort 172; other DMTs cohort 75). Overall, the mean age of patients was 36.5 years, 64.4% were women and ~90% were Caucasians. At baseline, mean MS duration since diagnosis was 7.2 years in the fingolimod and 4.8 years in the other DMTs cohorts. Overall, mean changes in MusiQoL index scores were −2.1 in the fingolimod cohort and −0.7 in the other DMTs cohort at Month 12, but improvement was not significant vs. baseline in both cohorts. Proportion of relapse-free patients increased significantly during the study vs. 0–12 months before the study in the fingolimod cohort (80.2% vs. 24.4%; *p* < 0.0001). Proportion of patients free from disability progression was 86.5% in the fingolimod cohort. The incidences of AEs were 59.9% and 50.6% in the fingolimod and other DMTs cohorts, respectively. First-dose monitoring of fingolimod observed no cases of symptomatic bradyarrhythmia. Three cases of bradycardia were reported in the fingolimod cohort: one after the first dose and two during the study. No cases of macular oedema were observed during the study.

**Conclusions:**

Fingolimod treatment maintained QoL over 12 months and was effective in reducing relapse rate and disability progression. No new safety findings were observed in this real-world observational study in Middle Eastern countries.

**Electronic supplementary material:**

The online version of this article (doi:10.1186/s12883-017-0913-3) contains supplementary material, which is available to authorized users.

## Background

Multiple sclerosis (MS), a chronic, auto-immune disease of the central nervous system (CNS), is characterised by inflammation, demyelination and axonal/neuronal destruction, which may lead to residual disability [1, 2]. Approximately 2.5 million people worldwide are affected with MS [3]. The prevalence of MS is increasing in the Middle Eastern countries, probably due to the influence of lifestyle changes from Western countries and environmental and genetic factors [4, 5]. The overall prevalence of MS in this region is 51.52/100000, with the female/male ratio ranging from 0.8 to 4.3 and an overall mean age at disease onset of 28.54 years [4].

Several disease-modifying treatments (DMTs) exist for MS, i.e. drugs that have the potential to modify or change the course of MS by acting on its underlying pathophysiology [6]. Fingolimod (FTY720, Gilenya®) is a first-in-class, oral sphingosine-1-phosphate (S1P) receptor immunomodulator that acts as a functional antagonist by internalising activated receptors [7].

Fingolimod has been approved in several countries for treatment of patients with relapsing forms of MS. The three large Phase 3 clinical trials of fingolimod—FREEDOMS [8], FREEDOMS II [9] and TRANSFORMS [10]—showed a significant reduction in relapse rate, magnetic resonance imaging-related lesion counts and brain volume loss vs. placebo and interferon β-1a in patients with relapsing-remitting MS (RRMS). These effects were sustained in the respective extension studies [11, 12], as reflected by low levels of MS disease activity and disability progression. Moreover, several observational studies reported that treatment with fingolimod showed improvement in patients’ quality of life (QoL) and satisfaction [13–20].

The safety and efficacy of fingolimod in MS patients have been established in clinical development programmes [8–12] as well as in a few non-interventional observational studies [21–26].

It is essential to assess the health-related QoL (HRQoL) outcome in patients with MS and evaluate the impact of treatments and care management in these patients. In 2008, Simeoni and colleagues developed the MS International QoL (MusiQoL) specifically to account for patients’ viewpoint on the impact of disease on their daily life and assess patient-reported HRQoL [27], which has been globally accepted by physicians. The importance of HRQoL outcome in the management of patients with MS using MusiQoL was also emphasised and recommended by the Middle East MS Advisory Group as part of routine care [28]. However, evidence on the use of fingolimod in real-world clinical practice in countries such as the Middle East is limited, and data are sparse on patient-reported HRQoL, particularly using the MS-specific MusiQoL questionnaire.

The present **P**rospective **E**valuation of T**r**eatment with **F**ingolim**o**d fo**r M**ultiple **S**clerosis (PERFORMS) non-interventional study was conducted to assess the HRQoL of RRMS patients and expand the knowledge of fingolimod effectiveness and safety in real-world clinical practice, primarily in the Middle Eastern countries. The objectives of the present study were to explore the effect of fingolimod on patients’ HRQoL in relation to other DMTs, assess the effectiveness of fingolimod in relation to other DMTs, assess the incidence of selected safety outcomes, describe the overall safety profile of fingolimod and describe physicians’ impression of treatment with fingolimod in routine clinical practice.

## Methods

### Patient population

Men and women aged ≥18 years who were diagnosed with RRMS and were started on MS therapy with fingolimod or other approved DMTs within 4 weeks prior to study entry and who provided written informed consent were included in the study. The MS therapy was part of the patients’ routine medical care and was prescribed in compliance with the local prescribing information. In countries where fingolimod was approved as a second-line therapy, only patients who had switched from MS treatment to either fingolimod or other DMTs within 4 weeks prior to study entry were included.

Patients with contraindications mentioned in the local prescribing information for the treatment were not included in the study.

### Study design

This was a 12-month, observational, multicentre, prospective-cohort, real-world study. The study was conducted in 27 outpatient centres across Egypt, Israel, Kuwait, Lebanon, United Arab Emirates, Saudi Arabia and Thailand from March 2012 to January 2015. Patients with RRMS were enrolled at a ratio of 2:1 (fingolimod:other DMTs) to obtain more data on fingolimod (hereafter, fingolimod cohort refers to patients taking fingolimod at study entry), while additionally obtaining data in a parallel cohort (hereafter, other DMTs cohort refers to patients taking another MS DMT at study entry). This ratio was controlled primarily at the investigator site level and secondarily at the country level. The choice of MS treatment was made within the context of the patient’s routine medical care and independent of the decision to include the patient in the study.

Data collected for the study originated from the routine care of patients and were recorded by physicians at study entry (baseline) and at Months 3, 6 and 12. Completion of the MusiQoL questionnaire by patients and the Clinical Global Impression-Improvement scale (CGI-I) by physicians were the only study-specific requirements. No additional visits or diagnostic or monitoring procedures were mandated by the protocol. Due to the observational nature of the study, no neuroimaging assessments were mandated and central reading was not performed.

### Study outcomes and endpoints

#### Effectiveness

##### Health-related quality of life

Patient-reported HRQoL was assessed at baseline and at Months 6 and 12 using MusiQoL. This multidimensional (nine dimensions) self-administered questionnaire consists of 31 items, with responses describing frequency/extent of an event on a 5-point scale ranging from 1 (never/not at all) to 5 (always/very much) [27]. If a patient changed or discontinued the medication of interest (MOI), then the questionnaire was requested to be completed at the time of the MS therapy change. The change in MusiQoL scores from baseline to Months 6 and 12 was reported in the study.

##### Physician impression of treatment

At the study completion, physicians were asked to provide a subjective evaluation of the improvement of patients over the study period using the CGI-I. The CGI-I is a 7-point Likert-type scale, allowing physicians to rate the change in the patient’s condition over time (from ‘very much improved’ to ‘very much worse’) and has been a robust tool for physicians, accounting for both therapeutic efficacy and treatment-emergent adverse events (AEs) response/rates [29].

##### Multiple sclerosis relapses

MS relapses were reported according to the physician’s judgement, with the recommendation to apply the international definition of a relapse [30]. The proportion of patients with MS relapses at 12–24 months and 0–12 months prior to study and during the 12 months of study duration was reported. Kaplan-Meier plot was provided to report time to the first relapse during the study.

##### Disability

Neurologic disability was measured by the Expanded Disability Status Scale (EDSS) score [31]. Disability progression was determined according to the baseline severity of symptoms and based on previously used criteria [32, 33], and was defined as a sustained increase in the EDSS score by 1 point if baseline EDSS was ≤5.0 or by 0.5 points if baseline EDSS was ≥5.5. The change in EDSS scores from baseline to Months 6 and 12/end of study (EOS) and proportions of patients free from disability progression at Months 6 and 12/EOS were reported.

Disability was also assessed by reporting patients’ walking ability. Physicians used the four-level Likert-type measure to determine whether the patient was able to walk unrestricted/unable to walk unrestricted but no assistive device used/unable to walk unrestricted and assistive device used/unable to walk at all. Patients’ walking ability at baseline and Months 6 and 12 was reported.

#### Safety

Safety assessments consisted of collecting all AEs and serious AEs (SAEs) and assessing their severity and relationship to the study drug. Clinically significant abnormalities in haematology and clinical chemistry were reported. The proportion of patients with AEs, SAEs, AEs leading to treatment discontinuation and selected AEs (such as symptomatic bradyarrhythmia, macular oedema, increase in liver enzymes and infections) by Month 12/EOS were reported. Ophthalmic examinations were performed at each time point, including the presence of macular oedema and the assessment of visual acuity for both eyes.

First-dose monitoring of fingolimod included haemodynamic assessments at several pre- and post-dose time points: sitting pulse (beats per minute, continuous variable) and blood pressure (mm Hg, continuous variable) per usual clinical practice. Additionally, any new incidence of bradycardia, new or worsening electrocardiography findings and the need for concomitant treatment were monitored at first fingolimod dose for the fingolimod cohort.

### Statistical analysis

The target sample size of 246 patients, with a 2:1 ratio (fingolimod:other DMTs), was determined empirically. All effectiveness outcomes were determined on the full analysis set (FAS), defined as patients who were assigned to either the fingolimod or the other DMTs cohort at baseline and remained in the same cohort (MOI) throughout the study as well as patients who switched cohort or discontinued the MOI but remained in the study up to Month 12. All the safety analyses were performed on the safety set, defined as the set of patients included in the analyses and who used fingolimod or other DMTs for at least 1 day and at any time during the study. The safety set considered patients who switched from their original cohort (from ‘fingolimod’ to ‘other DMTs’ or vice versa) during the study. The MOI was defined as the MS DMT initiated prior to study entry (baseline) or within a month prior to baseline.

The statistics were summarised descriptively in the study, except for the few comparisons performed in the two cohorts separately (no comparisons between cohorts). The mean MusiQoL (for each dimension and for the index score) and EDSS scores at Months 6 and 12/EOS vs. baseline were analysed using paired t-tests, providing 95% confidence intervals (CIs) of the mean difference and the *p* value for the test. The mean change in MusiQoL was calculated from baseline to Months 6 and 12/EOS. The proportion of patients with at least one MS relapse during the study vs. 0–12 months before study was analysed using a McNemar test for repeated measures. The time to first relapse was computed to provide Kaplan-Meier estimates. Missing data on drug discontinuation date and drug initiation date were imputed using the next drug initiation date and preceding drug date, respectively. Missing data in the self-reported MusiQoL were imputed as suggested by Simeoni and colleagues in 2008 [27]. To minimise the risk of self-selection bias, participating physicians were encouraged to enrol patients in both cohorts in a consecutive manner during a regular visit.

### Ethical and good clinical practice

The study protocol and amendment were approved by the Independent Ethics Committees and Institutional Review Boards for each centre per local regulations. All patients provided written informed consent before study entry. The study was conducted in compliance with the ethical principles of the Declaration of Helsinki and the International Conference on Harmonization Good Clinical Practice Guidelines [34].

## Results

### Patient disposition and baseline characteristics

Of the 249 enrolled patients (fingolimod cohort, 174; other DMTs cohort, 75), 247 were included in the FAS (fingolimod cohort, 172; other DMTs cohort, 75). Two patients in the fingolimod cohort were excluded from the FAS, as fingolimod was not newly initiated (within 4 weeks) prior to study entry. The safety set consisted of 177 patients in the fingolimod cohort and 87 in the other DMTs cohort. The majority of the patients (88.7%) completed the 12-month follow-up (Additional file 1: Table S1).

Patients’ demographics and baseline characteristics are described in Table 1. The overall mean age of patients was 36.5 years, 64.4% were women and Caucasians were predominant (~90%). At baseline, the mean duration since diagnosis of MS was 7.2 years in the fingolimod cohort and 4.8 years in the other DMTs cohort. Overall, 113 (65.7%) patients in the fingolimod cohort and 62 (82.7%) in the other DMTs cohort had at least one MS relapse in the previous year before study entry. The mean ± standard deviation (SD) number of relapses in the 12 months before study start was 1.1 ± 0.9 and 1.2 ± 0.8 in the fingolimod and other DMTs cohorts, respectively. Before study entry, the proportion of treatment-naïve patients was 14.0% in the fingolimod cohort and 61.3% in the other DMTs cohort. Among the patients who were on MS DMTs before the study, the majority in both cohorts were on interferon β therapies (Table 1). At the study start, most of the patients in the other DMTs cohort (~73%) were prescribed interferon β therapies, followed by natalizumab (20.0%) and glatiramer acetate (6.7%).

**Table 1:** Patient demographics and baseline characteristics

	Fingolimod cohort *N* = 172	Other DMTs cohort *N* = 75	Total *N* = 247
Age (years)			
Mean	36.7 ± 11.2	36.2 ± 12.2	36.5 ± 11.5
Median (min–max)	35.0 (18.0–64.0)	34.0 (18.0–68.0)	34.0 (18.0–68.0)
Women, n (%)	112 (65.1)	47 (62.7)	159 (64.4)
BMI (kg/m^2^), n (%)			
Overweight (25 ≤ BMI < 30)	47 (27.3)	14 (18.7)	61 (24.7)
Obese (BMI ≥30)	18 (10.5)	12 (16.0)	30 (12.1)
Race, n (%)			
Caucasian	153 (89.0)	68 (90.7)	221 (89.5)
Asian	4 (2.3)	1 (1.3)	5 (2.0)
Other	15 (8.7)	6 (8.0)	21 (8.5)
MS disease history			
Duration since MS diagnosis (years)			
Mean	7.2 ± 6.1	4.8 ± 6.8	6.5 ± 6.4
Median (min–max)	5.3 (0.0–32.0)	1.2 (0.0–23.9)	4.2 (0.0–32.0)
Duration since the first MS symptoms (years)			
Mean	9.4 ± 7.6	7.5 ± 9.1	8.9 ± 8.1
Median (min–max)	7.3 (0.1–34.7)	3.2 (0.0–44.1)	6.6 (0.0–44.1)
Duration since the most recent MS relapse (months)			
Mean	10.4 ± 14.8	6.1 ± 8.6	9.0 ± 13.2
Median (min–max)	5.0 (0.0–87.0)	3.0 (0.0–50.0)	4.0 (0.0–87.0)
Number of MS relapses in the 12 months before baseline			
Mean	1.1 ± 0.9	1.2 ± 0.8	1.1 ± 0.9
Median (min–max)	1.0 (0.0–5.0)	1.0 (0.0–3.0)	1.0 (0.0–5.0)
Number of MS relapses 12–24 months before baseline			
Mean	0.9 ± 1.1	0.5 ± 0.8	0.8 ± 1.0
Median (min–max)	1.0 (0.0–5.0)	0.0 (0.0–3.0)	0.0 (0.0–5.0)
History of MS patients before study, n (%)			
Treatment-naïve patients ^a^	24 (14.0)	46 (61.3)	-
Patients on any approved MS DMT	148 (86.0)	29 (38.7)	-
Type of DMTs prescribed before study entry, n (%)			
Fingolimod	8 (5.4)	4 (13.8)	-
Any interferon β	103 (69.6)	15 (51.7)	-
Glatiramer acetate	17 (11.5)	8 (27.6)	-
Natalizumab	16 (10.8)	2 (6.9)	-
Other	4 (2.7)	0 (0.0)	-

Data are presented as mean ± SD, unless stated otherwise; percentages were calculated based on the total number of patients in each treatment cohort (n)

*BMI* body mass index, *DMT* disease-modifying treatment, MS multiple sclerosis, *min* minimum, *max* maximum, *SD* standard deviation

^a^Treatment-naïve patients are patients who had never received any MS DMT before study entry (±4 weeks)

### Drug exposure

The mean duration of drug exposure during the study was 321.8 ± 147.7 days (151.6 patient-years) in the fingolimod cohort and 337.6 ± 117.0 days (69.3 patient-years) in the other DMTs cohort.

### Effectiveness

#### Health-related quality of life

Overall, >97% patients completed the MusiQoL questionnaire at baseline. At Month 12/EOS, 94.6% patients completed this questionnaire in the fingolimod cohort and 87.7% in the other DMTs cohort.

**Fig. 1:**
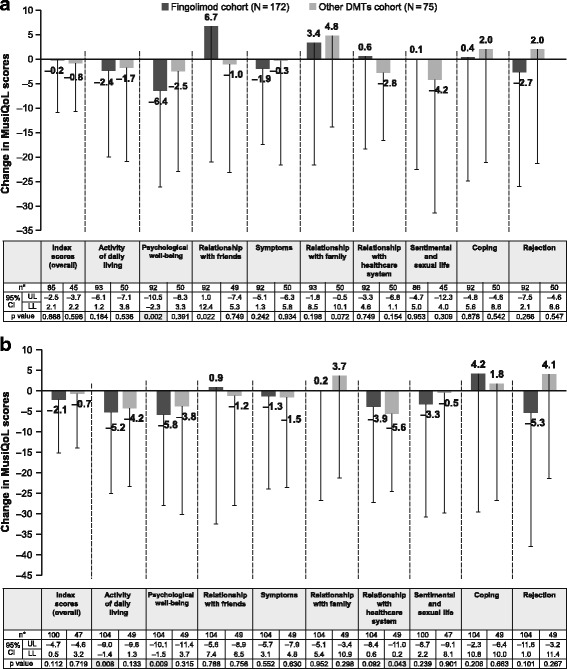
Mean (±SD) change in MusiQoL scores from baseline to Months (**a**) 6 and (**b**) 12 (FAS). *No. of pairs. Displayed is the difference in mean between the baseline score and the score of the evaluation time (a negative difference indicates a QoL improvement and a positive difference indicates a QoL deterioration). *DMT, disease-modifying treatment; CI, confidence interval; FAS, full analysis set; LL, lower limit; MusiQoL, Multiple Sclerosis International Quality of Life; QoL, quality of life; SD, standard deviation; UL, upper limit*

During the study, overall mean change (CI; *p* value) in the MusiQoL index score was -0.2 [−2.5 to 2.1; *p* = 0.868] at Month 6 and −2.1 [−4.7 to 0.5; *p* = 0.112] at Month 12 for the fingolimod cohort and −0.8 [−3.7 to 2.2; *p* = 0.598] at Month 6 and −0.7 [−4.6 to 3.2; *p* = 0.719] at Month 12 for the other DMT cohort, but the improvement was not statistically significant vs. baseline in both cohorts (Fig. 1). The fingolimod cohort showed significant improvements in MusiQoL sub-scores of −6.4 (−10.5 to −2.3; *p* = 0.002) for the ‘psychological well-being’ dimension at Month 6 and −5.2 (−9.0 to −1.4; *p* = 0.008) for the ‘activity of daily living’ and −5.8 (−10.1 to −1.5; *p* = 0.009) for ‘psychological well-being’ dimensions at Month 12 (both *p* < 0.01 vs. baseline). There was a significant improvement in the sub-score for the ‘relationship with healthcare system’ dimension at Month 12 (−5.6 [−11.0 to −0.2]; *p* = 0.043 vs. baseline) in the other DMTs cohort.

The questions to patients under the ‘psychological well-being’ dimension included if they felt anxious; felt depressed or gloomy; felt like crying; or felt nervous or irritated by a few things or situations. The questions to patients under the ‘activity of daily living’ dimension included if they had difficulty walking or moving outside; difficulty with outdoor activities, i.e. shopping, going out to a movie, etc.; difficulty walking or moving around at home; been troubled by their balance or walking problems; difficulty with leisure activities at home, i.e. do-it-yourself, gardening, etc.; difficulty with their occupational activities, i.e. integration, interruption, limitation, been quickly tired, etc.; or been short of energy. The questions to patients under the ‘relationship with healthcare system’ dimension included if they were satisfied with the information on their disease or the treatment given by the doctors, nurses, psychologists taking care of their MS; felt understood by the doctors, nurses, psychologists taking care of their MS; or were satisfied with their treatments [28].

#### Physician impression of treatment

**Fig. 2:**
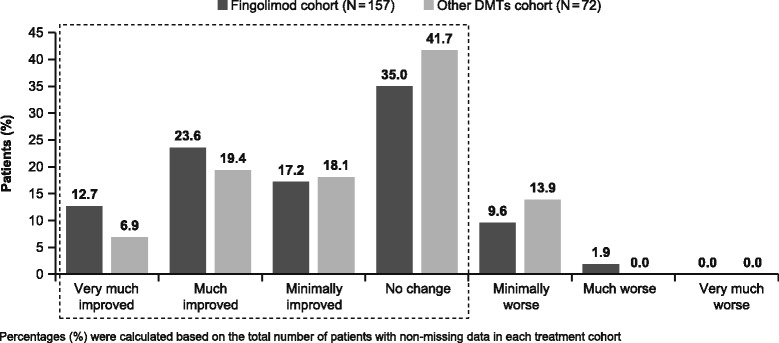
Clinical global impression on MS improvement from baseline to EOS by treatment (FAS). *DMT, disease-modifying treatment; EOS, end of the study; FAS, full analysis set; MS, multiple sclerosis*

The CGI-I score was completed in >90% of patients at EOS. Physicians indicated that 88.5% of patients in the fingolimod cohort and 86.1% in the other DMTs cohort showed either improvement or no change in MS on the CGI-I scale (Fig. 2).

#### MS relapses

**Fig. 3:**
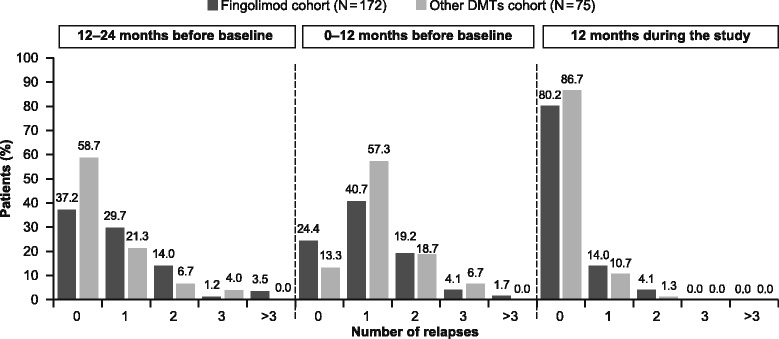
Proportion of patients with relapses in the 12–24 and 0–12 months before baseline and during the study by treatment (FAS). *DMT, disease-modifying treatment; FAS, full analysis set*

**Fig. 4:**
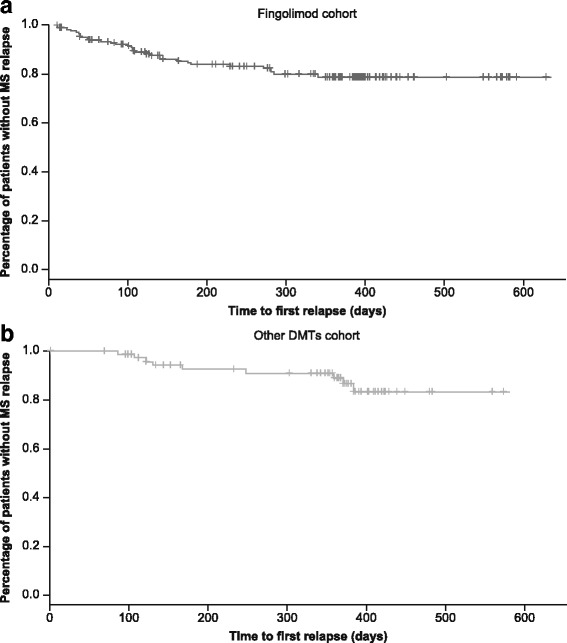
Survival curves of time to the first MS relapse during the study (Kaplan-Meier plot; FAS). **a** Fingolimod cohort. **b** Other DMTs cohort. *DMT, disease-modifying treatment; FAS, full analysis set; MS, multiple sclerosis*

The majority of patients (>80%) experienced no relapses during the study. The proportion of relapse-free patients increased significantly (*p* < 0.0001) during the study vs. 0–12 months before the study in both cohorts (Fig. 3). The mean number of relapses during the study was 0.2 ± 0.5 in the fingolimod cohort and 0.1 ± 0.4 in the other DMTs cohort. The survival curve of time to the first MS relapse during the study is depicted in Fig. 4. Mean duration to the first MS relapse was >4 months in the fingolimod cohort (123.1 ± 92.3 days) and >7 months in the other DMTs cohort (218.8 ± 122.5 days).

#### Disability

**Fig. 5:**
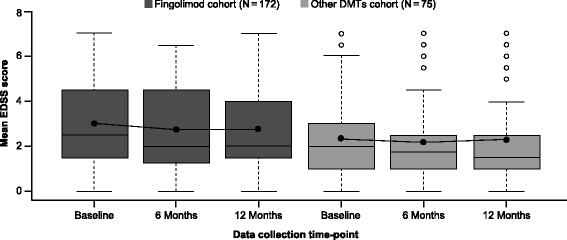
Box and whiskers plot for mean EDSS score during the study by time point (FAS). *DMT, disease-modifying treatment; EDSS, Expanded Disability Status Scale; FAS, full analysis set*. The box plot’s horizontal lines represent the 25th percentile, median and the 75th percentile of the mean EDSS score for top, middle and bottom lines, respectively. The whiskers represent the magnitude from the 10th to the 90th percentiles. The dots on the chart represent mean values across the study period. Outlier values are represented with empty circles

**Fig. 6:**
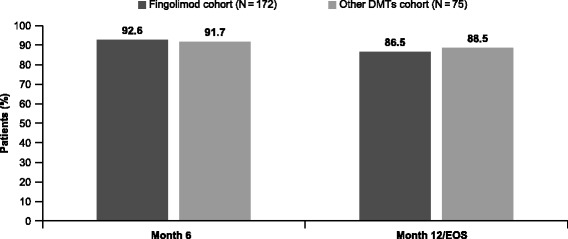
Proportion of patients free from disability progression according to EDSS score (FAS). *DMT, disease-modifying treatment; EDSS, Expanded Disability Status Scale; EOS, end of study; FAS, full analysis set*

Mean EDSS scores improved significantly from baseline (3.0 ± 1.7) to Month 6 (2.7 ± 1.9, *p* < 0.05) in the fingolimod cohort and was maintained up to Month 12/EOS (2.7 ± 1.8, *p* = 0.614). There were no significant improvements in the EDSS scores from baseline (2.3 ± 1.7) to Months 6 (2.2 ± 1.8, *p* = 0.391) and 12 (2.3 ± 1.8, *p* = 0.424) in the other DMTs cohort (Fig. 5). The proportion of patients free from disability progression was 86.5% in the fingolimod cohort and 88.5% in the other DMTs cohorts over 12 months (Fig. 6).

#### Walking ability

The proportions of patients able to walk or not, with or without using an assistive device, over 12 months during the study are summarised in Table 2. At Month 12/EOS, 75.7% patients in the fingolimod cohort (baseline, 73.3%) and 84.2% in the other DMTs cohort (baseline, 88.0%) were able to walk unrestricted outside home. Overall, 1.8% of the patients were unable to walk at Month 12/EOS during the study.

**Table 2:** Patients’ walking ability over 12 months of study period (FAS)

Walking ability, n (%)	Fingolimod cohort *N* = 172			Other DMTs cohort *N* = 75		
	Baseline *n* = 172	Month 6 *n* = 134	Month 12/EOS *n* = 111	Baseline *n* = 75	Month 6 *n* = 63	Month 12/EOS *n* = 57
Patients with walking ability assessment	165 (95.9)	112 (83.6)	111 (100.0)	75 (100.0)	59 (93.7)	57 (100.0)
Walking ability assessment						
Unable to walk	7 (4.2)	1 (0.9)	2 (1.8)	6 (8.0)	4 (6.8)	1 (1.8)
Not able to walk unrestricted outside home (assistive device used)	16 (9.7)	16 (14.3)	15 (13.5)	0 (0.0)	3 (5.1)	4 (7.0)
Not able to walk unrestricted outside home (assistive device not used)	21 (12.7)	15 (13.4)	10 (9.0)	3 (4.0)	1 (1.7)	4 (7.0)
Able to walk unrestricted outside home	121 (73.3)	80 (71.4)	84 (75.7)	66 (88.0)	51 (86.4)	48 (84.2)

Data are presented herewith as n (%)

*DMT* disease-modifying treatment, *EOS* end of study, *FAS* full analysis set

### Safety

The incidence of AEs was 59.9% in the fingolimod cohort and 50.6% in other DMTs cohort (Table 3). The most commonly occurring AEs were MS relapse (10.7%), lymphopaenia (7.9%) and increase in liver enzymes (6.8%) in the fingolimod cohort and MS relapse (8.0%), fatigue (6.9%) and gait disturbance (6.9%) in the other DMTs cohort. The most frequently (≥5%) observed abnormal blood counts were lymphopaenia (*n* = 14, 7.9%), increase in hepatic enzymes (*n* = 12, 6.8%), leukopaenia (*n* = 9, 5.1%) and decrease in lymphocyte counts (*n* = 9, 5.1%) in the fingolimod cohort.

**Table 3:** Incidence of AEs (safety set)

	Fingolimod cohort *N* = 177	Other DMTs cohort *N* = 87
Safety profile, n (%)		
Patients with any AE	106 (59.9)	44 (50.6)
Patients with any AE leading to drug discontinuation	29 (16.4)	5 (5.7)
Patients with an SAE	10 (5.6)	1 (1.1)
Deaths	0 (0.0)	0 (0.0)
Most frequent AEs (≥3% of patients for any group; n [%]; preferred terms)		
MS relapse	19 (10.7)	7 (8.0)
Lymphopaenia	14 (7.9)	0 (0.0)
Hepatic enzyme increased	12 (6.8)	0 (0.0)
Fatigue	11 (6.2)	6 (6.9)
Muscular weakness	10 (5.6)	5 (5.7)
Leukopaenia	9 (5.1)	3 (3.4)
Lymphocyte count decreased	9 (5.1)	0 (0.0)
Dizziness	8 (4.5)	2 (2.3)
Gait disturbance	7 (4.0)	6 (6.9)
MS worsening	4 (2.3)	4 (4.6)
Pain in extremity	6 (3.4)	2 (2.3)
Headache	4 (2.3)	3 (3.4)
Micturition urgency	3 (1.7)	3 (3.4)
Influenza	0 (0.0)	4 (4.6)
Paraesthesia	0 (0.0)	4 (4.6)
Selected AEs, n (%)		
Symptomatic bradyarrhythmia	0 (0.0)	0 (0.0)
Macular oedema	0 (0.0)	0 (0.0)
Increase in liver enzymes	14 (8.9)	2 (2.8)
Any infection	4 (2.5)	2 (2.8)
Other (any other AE)	50 (31.6)	28 (38.9)

Safety set: all AEs are reported in patients on MOI or after a switch; if no medication is taken at the time of the AE start, the AE will be reported under the category of the medication taken within the last 45 days

*AE* adverse event, *DMT* disease-modifying treatment, *MOI* medication of interest, *MS* multiple sclerosis, *SAE* serious adverse event

Approximately 16.0% (*n* = 29) of the patients discontinued the treatment due to AEs in the fingolimod cohort and 5.7% (*n* = 5) in the other DMTs cohort. The most frequent AEs (≥2% of patients in any cohort) leading to treatment discontinuation were decrease in lymphocyte count (*n* = 6, 3.4%) and MS relapse (*n* = 4, 2.3%) in the fingolimod cohort and pain in extremity (*n* = 2, 2.3%) in the other DMTs cohort.

The percentage of patients experiencing SAEs was 5.6% (*n* = 10) in the fingolimod cohort and 1.1% (*n* = 1) in the other DMTs cohort. Two cases each of lymphopaenia, leukopaenia and MS relapse were reported; the remaining events were singular and diverse in nature in the fingolimod cohort. One case each of leukopaenia and neutropaenia were reported in the other DMTs cohort. Further details of treatment discontinuation and SAEs are provided in Additional file 2: Table S2. No deaths occurred during the study.

The first-dose monitoring of fingolimod-treated patients showed a minor and transient decrease in pulse rate and blood pressure. One patient reported symptomatic bradycardia and one patient returned for monitoring with new or worsening electrocardiogram findings after the first dose of fingolimod. There were three cases of bradycardia reported in total in the fingolimod cohort during the study. No cases of symptomatic bradyarrhythmia or macular oedema were reported in either cohort (Table 3).

## Discussion

The present observational PERFORMS study explored the real-world experience of fingolimod treatment in patients with RRMS in Middle Eastern countries. The study reported that QoL was maintained over 12 months in patients with RRMS in the fingolimod cohort. Fingolimod was effective in reducing the relapse rate and disability progression. The results from this real-world study are consistent with the efficacy and safety profile of fingolimod established in clinical trials [8–12].

Considering the observational nature of the study, no formal statistical comparison was performed; however, patients’ sociodemographics, such as distribution of age, gender and race, were similar in both cohorts. These characteristics were comparable to those of patients with RRMS in the previous observational study in Kuwait [35] and also consistent with characteristic of patients included in the large randomised FREEDOMS, FREEDOMS II and TRANSFORMS studies [8–12].

In terms of disease history, the mean duration since MS diagnosis was longer in the fingolimod cohort than in the other DMTs cohort at baseline. This was further reflected with the fact that ~50% of the fingolimod cohort had the first MS symptoms >5 years prior to study start, as opposed to the other DMTs cohort, where 50% of patients had the first diagnosis <15 months prior to study start. In addition, the percentage of patients switching from prior natalizumab to fingolimod treatment was high at study entry. Moreover, the mean baseline EDSS scores were higher and treatment-naïve patients were fewer in the fingolimod cohort compared with the other DMTs cohort. Patients included in the fingolimod cohort were thus more ‘chronic’ and had more ‘residual disability’ than those in the other DMTs cohort. Such imbalances in baseline characteristics between treatment groups are common in open-label, observational studies. It was reported that baseline EDSS scores significantly impact the treatment response with the DMTs in patients with RRMS [36]. These differences in baseline characteristics between groups, in particular the EDSS score, might have led to comparable effectiveness results between fingolimod and other DMTs cohorts in this study.

The overall MusiQoL index score was high in both cohorts during the study. In the fingolimod cohort, two dimensions showed significant improvement during the study: ‘activity of daily living’ and ‘psychological well-being’. However, these two dimensions were also the ones with the lowest scores at baseline, and the subsequent improvement in scores may actually reflect a regression to the mean effect [37]. The overall MusiQoL index score of 64.4 at EOS in the fingolimod cohort was in line with the previously presented 6-month interim analysis from the real-world VIRGILE study in France, where median MusiQoL scores ranged from 62.4 to 65.7 [13]. As observed in several observational studies using different questionnaires [13–20], the overall HRQoL with fingolimod remained stable over 12 months in the present study. In the other DMTs cohort, the ‘relationship with the healthcare system’ dimension significantly improved throughout the study period. The global HRQoL index showed no improvement.

In the study, overall, treating physicians considered that the clinical impression of 88.5% of fingolimod-treated patients either improved or had not changed. This is consistent with results observed in the 6-month open-label Evaluate Patient Outcomes, Safety, and Tolerability of Fingolimod (EPOC) study, where CGI-I scores were significantly lower in the fingolimod cohort vs. standard-of-care DMT cohort (3.2 vs. 3.9, respectively; *p* < 0.0001), indicating a greater perceived improvement [19].

In the fingolimod cohort, 31 (18.0%) patients experienced at least one relapse during the study. This result was in line with the 12-month randomised double-blind TRANSFORMS study reporting that ~20.0% of patients had at least one relapse [10], but was higher than the previously reported retrospective study using the US Claims Database where only ~13.0% of patients had at least one relapse over 360 days of treatment in the fingolimod group [21]. In this retrospective study, only 33% of patients had MS relapse within 1 year before study entry when switched from interferons to the fingolimod cohort at baseline [21], whereas the majority of the patients (70.9%) included in the current study had experienced at least one relapse within 1 year before study (mean duration since last relapse: 9 months). The results of the study therefore need to be evaluated with caution considering the patient population and disease history at baseline in the fingolimod cohort.

The proportion of relapse-free patients reported in the study (80.2%) was in line with the 12-month TRANSFORMS (82.5%) [10] and the multicentre post-marketing real-world study (88.1%) by Totaro and colleagues [25] in patients with RRMS. This finding was higher than those in large randomised studies in patients with RRMS: 24-month FREEDOMS—70.4% [8] and FREEDOMS II—71.5% [9].

According to the EDSS measure, 86.5% of the patients were free from any disability progression in the fingolimod cohort at the EOS, which was lower than that in the randomised controlled TRANSFORMS study, wherein 93.3% of patients (95% CI, 90.9%–95.8%) had no disability progression [10]. The proportion of patients free from disability progression in the present study was in line with the 24-month randomised FREEDOMS [8] and FREEDOMS II [9] studies as well as the 3-year interim analysis of the 5-year PANGAEA registry records data from Germany [24].

There were no new safety concerns during fingolimod first-dose monitoring. During the study, a total of three cases of symptomatic/treated bradycardia and no cases of bradyarrhythmia were reported. These are known class effects and have been noticed to resolve without therapeutic intervention in other clinical trials [38, 39]. In the current study, no case of macular oedema, which is an identified risk with fingolimod treatment [40], was reported in the fingolimod cohort.

The number of patients reporting a decrease in lymphocyte counts, which is a known pharmacodynamic therapeutic effect of fingolimod, was low (5.1%) in the fingolimod cohort and comparable to that in earlier safety reports [41, 42]. Of note, reductions in lymphocyte counts with fingolimod in the present study did not show an increase in the risk of infections and was consistent with the data reported earlier in clinical studies as well as in the post-marketing setting [41]. Safety results reported in the study were in line with integrated safety analysis [41] and long-term studies [42], reporting no increased risk of infections, malignancies or serious cardiovascular events with fingolimod.

Owing to the observational, non-blinded and non-randomised nature of the study, different biases could have obscured any true causal association. Systemic differences between treatments may exist, influenced by decisions of the treating physicians who assigned patients to different drugs based on disease severity, disease duration, presence of co-morbidities and other confounding factors (i.e. associated with the choice of treatment and treatment outcome). These differences, due to an indication/channelling bias [43], can confound the association between treatment and treatment outcome. Patients with a longer progression of the disease or patients refractory to other DMTs were more likely to receive fingolimod, which might have resulted in the underestimation of the effectiveness of fingolimod. Although the QoL was self-reported by the patients, the MusiQoL questionnaires were transcribed by the physician or the study staff, which might have resulted in the risk of information bias.

As PERFORMS was a real-world, observational study, no neuroimaging assessments were mandated and magnetic resonance imaging read outs were not evaluated via a central reading facility. Therefore, neuroimaging findings were not considered as an outcome to be assessed.

## Conclusion

The study concluded that the QoL was maintained over 12 months with fingolimod treatment. Fingolimod was effective in reducing relapse rate and disability progression, confirming favourable results as found in large randomised clinical trials. The first dose of fingolimod appeared to be safe, and no new safety findings were observed in the study.

## Additional files

Additional file 1: Table S1. Patient disposition. (DOCX 51 kb)

Additional file 2: Table S2. Incidence of AEs causing treatment discontinuation and SAEs (safety set). (DOCX 51 kb)

## Abbreviations

AE: Adverse event
CGI-I: Clinical Global Impression-Improvement scale
CI: Confidence interval
CNS: central nervous system
DMTs: Disease-modifying treatments
EDSS: Expanded Disability Status Scale
EOS: end of study
EPOC: Evaluate Patient Outcomes, Safety, and Tolerability of Fingolimod
FAS: Full analysis set
HRQoL: Health-related quality of life
MOI: Medication of interest
MS: Multiple sclerosis
MusiQoL: Multiple Sclerosis International Quality of Life
PERFORMS: **P**rospective **E**valuation of T**r**eatment with **F**ingolim**o**d fo**r M**ultiple **S**clerosis
QoL: Quality of life
RRMS: Relapsing-remitting multiple sclerosis
S1P: Sphingosine-1-phosphate
SAE: Serious adverse event
SD: Standard deviation
