# Supplementary material for: Effectiveness, safety and health-related quality of life of multiple sclerosis patients treated with fingolimod: results from a 12-month, real-world, observational PERFORMS study in the Middle East
Source: BMC Neurol. 2017 Aug 7;17:150. doi: 10.1186/s12883-017-0913-3 (PMC5547540; doi:10.1186/s12883-017-0913-3)
Supplement: Supplementary file 1 — Patient disposition. (DOCX 51 kb) [file 12883_2017_913_MOESM1_ESM.docx]

**Additional file 1: Table S1.** Patient disposition

| **Patient disposition, n (%)** | **Fingolimod cohort** | **Other DMTs cohort** | **Total** |
| --- | --- | --- | --- |
| **Total patients included in the FAS** | 172 (69.6) | 75 (30.4) | 247 (100.0) |
| Patients who completed the study | 150 (87.2) | 69 (92.0) | 219 (88.7) |
| Patients who discontinued the study before 12 months | 22 (12.8) | 6 (8.0) | 28 (11.3) |
| Consent withdrawal | 3 (1.7) | 0 (0.0) | 3 (1.2) |
| Lost to follow-up | 18 (10.5) | 6 (8.0) | 24 (9.7) |
| Missing | 1 (0.6) | 0 (0.0) | 1 (0.4) |
| **Not on MOI throughout the study** | 42 (24.5) | 12 (16.0) |  |
| **Total patients who switched treatment during the study** | 6 (3.5) | 3 (4.0) | 9 (3.6) |
| Switched from other DMTs to fingolimod | - | 3 (4.0) | - |
| AE/lab or test abnormality | - | 1 (1.3) | - |
| Lack of efficacy | - | 1 (1.3) | - |
| Physicians’ decision | - | 0 (0.0) | - |
| Other | - | 1 (1.3) |  |
| Switched from fingolimod to other DMTs | 6 (3.5) | - | - |
| AE/lab or test abnormality | 3 (1.7) | - | - |
| Lack of efficacy | 1 (0.6) | - | - |
| Physicians’ decision | 2 (1.2) | - | - |
| Other | 0 |  |  |
| **Total patients who discontinued treatment during the study** | 36 (20.9) | 9 (12.0) | 45 (18.2) |
| AE/lab or test abnormality | 22 (12.9) | 5 (6.7) | - |
| Lack of efficacy | 2 (1.2) | 1 (1.3) | - |
| Physicians’ decision | 2 (1.2) | 2 (2.7) | - |
| Other | 8 (4.7) | 1 (1.3) | - |
| Missing | 2 | - |  |
| **Total patients included in the safety set** | 177 (67.0) | 87 (33.0) | 264 (100.0) |
| MOI throughout the study, n | 172 | 75 | - |
| Switched from other DMTs to fingolimod, n | 3 | - | - |
| Switched from fingolimod to other DMTs, n | - | 6 | - |
| Treatment changed, n | 2 | 6 | - |
| AE, adverse event; DMTs, disease modifying treatments; FAS, full analysis set; lab, laboratory; MOI, medication of interest | | | |
