# Supplementary material for: Effectiveness, safety and health-related quality of life of multiple sclerosis patients treated with fingolimod: results from a 12-month, real-world, observational PERFORMS study in the Middle East
Source: BMC Neurol. 2017 Aug 7;17:150. doi: 10.1186/s12883-017-0913-3 (PMC5547540; doi:10.1186/s12883-017-0913-3)
Supplement: Supplementary file 2 — Incidence of AEs causing treatment discontinuation and SAEs (safety set). (DOCX 51 kb) [file 12883_2017_913_MOESM2_ESM.docx]

**Additional file 2: Table S2.** Incidence of AEs causing treatment discontinuation and SAEs (safety set)

| **Patients, n (%)** | **Fingolimod cohort**  **N=177** | **Other DMTs cohort**  **N=87** |
| --- | --- | --- |
| **Patients with any AE leading to drug discontinuation** | **29 (16.4)** | **5 (5.7)** |
| Leukopaenia | 1 (0.6) | 0 (0.0) |
| Lymphocytosis | 0 (0.0) | 1 (1.1) |
| Neutropaenia | 1 (0.6) | 1 (1.1) |
| Bradycardia | 2 (1.1) | 0 (0.0) |
| Fatigue | 1 (0.6) | 0 (0.0) |
| Gait disturbance | 1 (0.6) | 0 (0.0) |
| Injection site rash | 0 (0.0) | 1 (1.1) |
| Laceration | 1 (0.6) | 0 (0.0) |
| Scratch | 1 (0.6) | 0 (0.0) |
| Gamma-glutamyltransferase increased | 1 (0.6) | 0 (0.0) |
| Haemoglobin decreased | 1 (0.6) | 0 (0.0) |
| Hepatic enzyme increased | 2 (1.1) | 0 (0.0) |
| Hormone level abnormal | 1 (0.6) | 0 (0.0) |
| Liver function test | 1 (0.6) | 0 (0.0) |
| Lymphocyte count decreased | 6 (3.4) | 0 (0.0) |
| Total lung capacity decreased | 1 (0.6) | 0 (0.0) |
| Weight increased | 1 (0.6) | 0 (0.0) |
| White blood cell count decreased | 1 (0.6) | 0 (0.0) |
| Back pain | 1 (0.6) | 0 (0.0) |
| Muscular weakness | 1 (0.6) | 0 (0.0) |
| Pain in extremity | 1 (0.6) | 2 (2.3) |
| Fibroadenoma of breast | 1 (0.6) | 0 (0.0) |
| Balance disorder | 1 (0.6) | 0 (0.0) |
| Dizziness | 2 (1.1) | 0 (0.0) |
| Headache | 0 (0.0) | 1 (1.1) |
| MS worsening | 0 (0.0) | 1 (1.1) |
| MS relapse | 4 (2.3) | 0 (0.0) |
| Muscular weakness | 1 (0.6) | 0 (0.0) |
| Syncope | 1 (0.6) | 0 (0.0) |
| Pregnancy | 3 (1.7) | 0 (0.0) |
| Amenorrhoea | 1 (0.6) | 0 (0.0) |
| Dyspnoea | 1 (0.6) | 0 (0.0) |
| Rash | 1 (0.6) | 0 (0.0) |
| **Patients with a SAE** | **10 (5.6)** | **1 (1.1)** |
| Leukopaenia | 2 (1.1) | 1 (1.1) |
| Lymphopaenia | 2 (1.1) | 0 (0.0) |
| Neutropaenia | 0 (0.0) | 1 (1.1) |
| Retinal detachment | 1 (0.6) | 0 (0.0) |
| Gait disturbance | 1 (0.6) | 0 (0.0) |
| Laceration | 1 (0.6) | 0 (0.0) |
| Muscular weakness | 1 (0.6) | 0 (0.0) |
| Grand mal convulsion | 1 (0.6) | 0 (0.0) |
| MS relapse | 2 (1.1) | 0 (0.0) |
| Partial seizures | 1 (0.6) | 0 (0.0) |
| Syncope | 1 (0.6) | 0 (0.0) |
| AE, adverse event; DMTs, disease modifying treatments; MS, multiple sclerosis; SAE, serious adverse event | | |
